# SIRT5-related desuccinylation modification of AIFM1 protects against compression-induced intervertebral disc degeneration by regulating mitochondrial homeostasis

**DOI:** 10.1038/s12276-023-00928-y

**Published:** 2023-01-18

**Authors:** Jianxin Mao, Di Wang, Dong Wang, Qi Wu, Qiliang Shang, Chu Gao, Huanbo Wang, Han Wang, Mu Du, Pandi Peng, Haoruo Jia, Xiaolong Xu, Jie Wang, Liu Yang, Zhuojing Luo

**Affiliations:** 1 Institute of Orthopedic Surgery, Xijing Hospital, Fourth Military Medical University, Xi’an, 710032 People’s Republic of China; 2 Intensive Care Unit, Heze Municipal Hospital, Heze, 274031 People’s Republic of China; 3 Medical Research Institute, Northwestern Polytechnical University, Xi’an, 710068 People’s Republic of China

**Keywords:** Diseases, Apoptosis, Post-translational modifications, Pathogenesis

## Abstract

Mitochondrial dysfunction plays a major role in the development of intervertebral disc degeneration (IDD). Sirtuin 5 (SIRT5) participates in the maintenance of mitochondrial homeostasis through its desuccinylase activity. However, it is still unclear whether succinylation or SIRT5 is involved in the impairment of mitochondria and development of IDD induced by excessive mechanical stress. Our 4D label-free quantitative proteomic results showed decreased expression of the desuccinylase SIRT5 in rat nucleus pulposus (NP) tissues under mechanical loading. Overexpression of *Sirt5* effectively alleviated, whereas knockdown of *Sirt5* aggravated, the apoptosis and dysfunction of NP cells under mechanical stress, consistent with the more severe IDD phenotype of *Sirt5* KO mice than wild-type mice that underwent lumbar spine instability (LSI) surgery. Moreover, immunoprecipitation-coupled mass spectrometry (IP-MS) results suggested that AIFM1 was a downstream target of SIRT5, which was verified by a Co-IP assay. We further demonstrated that reduced SIRT5 expression resulted in the increased succinylation of AIFM1, which in turn abolished the interaction between AIFM1 and CHCHD4 and thus led to the reduced electron transfer chain (ETC) complex subunits in NP cells. Reduced ETC complex subunits resulted in mitochondrial dysfunction and the subsequent occurrence of IDD under mechanical stress. Finally, we validated the efficacy of treatments targeting disrupted mitochondrial protein importation by upregulating SIRT5 expression or methylene blue (MB) administration in the compression-induced rat IDD model. In conclusion, our study provides new insights into the occurrence and development of IDD and offers promising therapeutic approaches for IDD.

## Disc degeneration: Boosting mitochondrial protein could ease lower back pain

Investigation of a mitochondrial protein linked with intervertebral disc degeneration (IDD) provides possible therapeutic targets for lower back pain. Intervertebral discs act as shock absorbers in the spine, and excessive load over time can cause disc degeneration and pain. Liu Yang and Zhuojing Luo at Fourth Military Medical University in Xi’an, China, and co-workers analyzed the effect of excessive mechanical load on protein expression in IDD samples from human patients and rodent models. The sirtuin 5 (SIRT5) protein helps maintain healthy mitochondria, a key factor in protecting against disc degeneration. The team found that SIRT5 expression was reduced in IDD, which affected the activity of another protein and directly led to mitochondrial dysfunction and disc degeneration. Upregulating SIRT5 alleviated compression-induced IDD in rat models. Targeting the protein may prove useful for future treatments.

## Introduction

Low back pain (LBP), a ubiquitous musculoskeletal disorder with high relapse and disability rates, affecting more than 500 million people globally, is one of the leading causes of health expenditure and financial burden^1,2^. As a multifactorial disease, the pathogenesis of LBP is not fully understood, but intervertebral disc degeneration (IDD) has been widely accepted as the primary pathological reason for LBP^3^. The intervertebral disc (IVD), composed of the central gelatinous nucleus pulposus (NP) and surrounding laminar annulus fibrosus (AF) and cartilage endplates (CEP), functions as a shock absorber of the spine to withstand mechanical impact. Accumulated evidence suggests that the degradation of extracellular matrix (ECM) and the apoptosis of cells within NP tissue, induced by sustained or excessive mechanical load, play a pivotal role in the pathogenesis of IDD^4–6^. However, the underlying molecular mechanism has not been fully elucidated.

As a highly dynamic organelle, mitochondria endow cells with the ability to adapt and respond to the ever-changing microenvironment through constant fusion and fission^7–10^. In addition to providing energy through oxidative phosphorylation, mitochondria are also involved in regulating reactive oxygen species (ROS), calcium handling, iron-sulfur cluster assembly (essential cofactors of many enzymes), and apoptosis^9,11–13^. Cells in NP tissue are believed to contain a few functional mitochondria, the dysfunction of which has been confirmed as a major contributor to IDD^5,14^. Although the causative role of mechanical stress in mitochondrial dysfunction within NP cells has been documented^6,15,16^, the limited understanding of pathogenesis and the lack of curative approaches for IDD indicate the need for further investigation of the underlying connections between the excessive mechanical load and the impairment of mitochondria within NP cells during the development of IDD.

Succinylation is a recently discovered post-translational modification of proteins that involves a reversible protein modification process during which succinyl-CoA is added to protein lysine groups. Succinylation usually causes alterations of protein‒protein electrostatic interactions due to the charge flip from positive to negative due to the succinyl adducts^17–19^. Since succinyl-CoA is produced in the mitochondria, succinylation is a fundamental biological process in regulating key metabolic processes within the mitochondria, such as the tricarboxylic acid (TCA) cycle, fatty acid metabolism, and ATP synthesis^18–20^. Interestingly, accumulating evidence strongly suggests succinylation modifications occur via a spontaneous, nonenzymatic mechanism, and the equilibrium of such a reversible modification process largely depends on the concentration of the reaction substrate, succinyl-CoA. Moreover, the extent of succinylation of a certain protein is precisely regulated by the desuccinylation process, which cannot be performed without the only known desuccinylase, mitochondria-located SIRT5^18^.

Increasing evidence has established that approaches targeting SIRT5 are fully capable of influencing the succinylation of proteins and could effectively suppress the occurrence and development of age-related degenerative diseases by maintaining mitochondrial homeostasis^18,21,22^. Recently, SIRT5-related desuccinylation modification has been proven to ameliorate heart failure by enhancing mitochondrial quality surveillance^23^. However, whether SIRT5 and desuccinylation modification of proteins participate in the regulation of compression-induced impairment of mitochondria in NP cells is worthy of further investigation.

In this study, we comprehensively analyzed the effect of excessive mechanical load on protein expression and demonstrated that SIRT5, the most reduced mitochondrial protein, played a protective role in compression-induced IDD. Moreover, we further revealed that dampened SIRT5 expression resulted in the increased succinylation of AIFM1, which in turn abolished the interaction between AIFM1 and CHCHD4, leading to mitochondrial dysfunction and finally contributing to the development of IDD under excessive mechanical load. Finally, we validated the efficacy of treatments targeting disrupted mitochondrial protein importation by upregulating SIRT5 expression or MB administration in the compression-induced rat IDD model, providing a new therapeutic strategy for IDD.

## Materials and methods

### Patient samples

NP specimens in this study were obtained from 18 patients (9 males and 9 females; mean age = 49.7 ± 12.3 years) with degenerative disc disease or scoliosis. The degree of IDD was assessed by three blinded spine surgeons according to a modified Pfirrmann grading system by magnetic resonance imaging (MRI). We classified Grade II (*n* = 4) and III (*n* = 6) samples as the Grade II/III group and Grade IV (*n* = 4) and V (*n* = 4) samples as the Grade IV/V group. Specimen data are shown in Supplementary Table 1. Ethical approval was obtained from the Institutional Review Board of Xijing Hospital of the Fourth Military Medical University (KY20203146-1), and informed consent was obtained from the donors. The study was conducted according to the Code of Ethics of the World Medical Association (Declaration of Helsinki).

### Animals

All animal experiments were approved by the Animal Use and Care Committee of the Fourth Military Medical University and conducted in accordance with the National Institute for Health Guide for the Care and Use of Laboratory Animals. Sirt5 knockout (KO) mice (C57BL/6 background) were purchased from the Shanghai Model Organisms Center. Mice were genotyped using the primer sets listed in Supplementary Table 2.

### Cell culture and transfection

Rat NP cells were cultured as described previously^5,24^. As the pressure measured in the human L4–L5 disc was 1.1 MPa for flexed-forward standing^25^, we selected 1 MPa as the excessive mechanical stress magnitude. Rat NP cells were subjected to 0 MPa or 1 MPa static compression stress for 0 h, 24 h, and 48 h, and we selected 24 h for the vast majority of experiments. siRNA transfection in vitro has been described previously^26^. The siRNA duplexes targeting rat *Sirt5, Aifm1, Chchd4*, and negative control (NC) were designed and synthesized by Tsingke Biotechnology, Beijing, China. The siRNA sequences used in this study are shown in Supplementary Table 3. Lentivirus-mediated gene transfer in vitro has been described previously^24^. Overexpression and knockdown efficacies were evaluated by qRT‒PCR or Western blotting.

### Rat tail compression (RTC) and histopathological analysis

RTC was used to induce IDD in 12-week-old male rats in this study^24^. Briefly, RTC was performed at the Co7–8 and Co8–9 levels with an adjustable mechanical loading apparatus. Rats were randomly divided into the compression group, lenti-*sirt5*-treated group, and MB-treated group after the operation (*n* = 5). Correspondingly, the rats in the sham group were only fitted with the apparatus without applying mechanical stress. The lenti-*sirt5*-treated group was intradiscally injected with lenti-*sirt5* (1 × 10^6^ TU), and the other three groups were injected with lenti-NC (1 × 10^6^ TU). The MB-treated group received an intraperitoneal injection of MB (2 mg/kg, twice a week)^27^, while the other three groups were injected with the same volume of normal saline. All animal experiments in this study followed the International Guiding Principles for Biomedical Research Involving Animals. The spine specimens were harvested 28 days after the operation. Then, the specimens were embedded in paraffin and sectioned (5 mm). Hematoxylin–eosin (HE) staining and safranin O-fast green (SO) staining were performed according to standard protocols, and the histological scores were evaluated by blinded assessment^5,24^.

### Lumbar spine (LSI) instability

An LSI model was used to induce IDD in 10-week-old male WT and *Sirt5* KO mice. Briefly, mice were anesthetized with isoflurane, and then, the supraspinous ligament, interspinous ligaments, and spinous process from lumbar 1 (L1) to lumbar 5 (L5) were resected. Correspondingly, in the sham group, only the paraspinal dorsal muscles from L1 to L5 were isolated. The IVDs of mice were harvested at 8 weeks post-operation. L4–L5 spines were processed for histological or histochemical staining.

### Four-dimensional label-free quantitative proteomics and IP-MS

The NP tissues were harvested from the RTC model, the NP tissues of the compressed IVDs were collected as the compression group, and the NP tissues of the adjacent IVDs above and below were collected as the control group (*n* = 3). To eliminate the influence of individual differences of rats as much as possible, we mixed the nucleus pulposus tissues of three rats as an independent sample. The differentially expressed proteins in the compression and control groups were analyzed by 4D label-free quantitative proteomics (PTM Bio, China).

For analysis of downstream targets of SIRT5, the coimmunoprecipitation products of SIRT5 in NP cells were separated by narrow-hole high-performance liquid chromatography. Mass spectral data were retrieved by MaxQuant (V1.6.6) software (Monitor Helix, China).

### Western blotting

Protein preparation and immunoblotting methods were described in previous studies^5^. The following antibodies were used: aggrecan (Millipore Sigma, AB1031, 1:1000), MMP13 (Abcam, ab39012, 1:1000), SIRT5 (Proteintech, 15122-1-AP, 1:500), TOM20 (Proteintech, 11802-1-AP, 1:1000), AIFM1 (Santa Cruz Biotechnology, sc-13116, 1:500), CHCHD4 (Proteintech, 21090-1-AP, 1:500), NDUFB8 (Complex I) (Abcam, ab192878, 1:2000), SDHB (Complex II) (Abcam, ab175225, 1:2000), UQCRC2 (Complex III) (Absin, abs116449, 1:2000), COX IV (Complex IV) (Cell Signaling Technology, 4844s, 1:2000), ATP5F1A (Complex V) (Abcam, ab176569, 1:2000), and succinylated lysine (PTM Bio, PTM-419, 1:500).

### Coimmunoprecipitation (Co-IP)

Co-IP experiments were performed to verify the binding between proteins of interest in NP cells. The co-IP complexes were purified by a MilliporeSigma^TM^ PureProteome^TM^ Protein A/G Mix Magnetic Bead System (#LSKAGAG10, Fisher Scientific, USA). The dosage of the IP antibody was 5 µg of anti-AIFM1 (Santa Cruz Biotechnology, sc-13116, USA) and 4 µg of anti-SIRT5 (Proteintech, 15122-1-AP, China). The proteins were harvested and analyzed by Western blot analysis.

### Oxygen consumption rate (OCR)

The OCRs for NP cells were detected using an Agilent Seahorse XFe24 Cell energy metabolism analyzer according to standard protocols^28^. NP cells were treated sequentially with 1 μM oligomycin (ATP synthetase inhibitor), 2 μM FCCP (uncoupling agent), and 1 μM rotenone & 1 μM antimycin A (electron transport chain inhibitors). Wave software and GraphPad Prism software were used to analyze the datasets. Basal OCR was calculated as [OCR_initial_ − OCR_R+A_]. The maximum respiration rate was computed as [OCR_FCCP_ − OCR_R+A_]. ATP production was calculated as [OCR_initial_ − OCR_Oligo_].

### Real-time quantitative RT‒PCR (RT‒qPCR)

Total RNA from rat NP cells was collected using a Total RNA Kit (Omega Biotek, Norcross, GA, USA) according to the manufacturer’s instructions. RNA concentrations were detected by the ratio of the absorbance at 260 nm to 280 nm. Then, PrimeScript RT Master Mix (TaKaRa, Japan) was used to reverse transcribe RNA to cDNA followed by quantitative PCR analysis using TB Green Premix Ex Taq II (TaKaRa, Japan). The reactions were performed with CFX96 (Bio-Rad, USA). The expression of the gene of interest was normalized to β-actin, and the fold change was calculated by the comparative threshold cycle method. The primers used in this study are shown in Supplementary Table 4.

### Apoptosis assay

Terminal deoxynucleotidyl transferase dUTP nick end labeling (TUNEL) staining was performed to detect apoptosis in rat IVD tissue sections and NP cells. The methods were described previously^5^. In short, the tissue sections and cell slides were stained with the One-Step TUNEL Apoptosis Assay Kit (Beyotime Biotechnology, China) according to the manufacturer’s instructions. Flow cytometry with Annexin V-FITC/PI was also performed to detect the apoptosis of NP cells. Treated cells were harvested with 0.25% trypsin and washed 3 times with cold PBS. Then, the cells were resuspended in binding buffer and detected by an Annexin VPE/PI Apoptosis Detection Kit (BD Biosciences, USA, Flow Cytometry).

### Immunofluorescence staining

Rat NP cell slides were first fixed with 4% paraformaldehyde for 15 min at room temperature. Then, the cell slides were permeabilized with 0.3% Triton X-100 (MP Biomedical, USA) for 15 min at room temperature. After blocking with QuickBlock™ Blocking Buffer for Immunol Staining (Beyotime Biotechnology, China) for 1 h, the cell slides were incubated with primary anti-SIRT5 (Proteintech, 15122-1-AP, 1:50), anti-MMP13 (Proteintech 18165-1-AP, 1:100), anti-aggrecan (Millipore Sigma AB1031, 1:100), anti-TOM20 (Proteintech, 11802-1-AP, 1:200), anti-AIFM1 (Santa Cruz Biotechnology, sc-13116, 1:20) and anti-CHEJ4 antibodies (Proteintech, 21090-1-AP, 1:50) at 4 °C overnight. Then, the corresponding FITC- or Cy3-conjugated secondary antibodies (1:200) were used to incubate the slides for 2 h in the dark at room temperature. Finally, the slides were incubated with 4’,6-diamidino-2-phenylindole (DAPI) (Beyotime Biotechnology, China) for 20 min in the dark at room temperature. After each step, the cells were washed 3 times with PBS for 5 min. Cell slides were then observed under a fluorescence microscope (BX53, Olympus, Japan) or fluorescence confocal microscope (A1R, Nikon, Japan), and the fluorescence intensity was quantified with ImageJ software.

### In vitro succinylation assay

AIFM1 was purified from NP cells and subjected to reactions containing succinylation buffer (20 mmol/L pH 8.0 HEPES, 1 mmol/L dithiothreitol, 1 mmol/L phenylmethyl sulfonyl fluoride, and 0.1 mg/mL BSA) and different concentrations of succinyl-CoA (S1129, Sigma-Aldrich)^29^. The reaction mixture was incubated for 15 min at 30 °C. Then, loading buffer was added to stop the reaction. Finally, proteins were harvested and analyzed by Western blot analysis.

### X-ray, micro-CT, and MRI assays

The X-ray, micro-CT, and MRI assay methods were described in a previous study^24^. The above imaging studies were performed before the rats (4 weeks after surgery of the tail compression model) and mice (8 weeks after surgery of the lumbar spine instability model) were sacrificed.

### Mitochondrial morphology analysis in vitro

The mitochondrial length and aspect ratio (AR, ratio between the major and minor axes) within NP cells (average value of all mitochondria in the cell; mean ± SD of three independent experiments) were determined using ImageJ software, as previously reported^30^.

### Measurement of intracellular ATP content

The ATP content of NP cells was measured using the ATP Bioluminescence Assay Kit (Beyotime Biotechnology, China) according to the manufacturer’s instructions.

### Statistical analysis

Data are presented as the mean ± standard deviation (SD). The histological scores and Pfirrmann grades of IVDs were analyzed using the Kruskal–Wallis h-test. Student’s t test or one-way ANOVA was used to analyze the differences between two or among multiple groups. All statistical analyses were performed with GraphPad Prism 9.0 software and SPSS 26.0. *p* < 0.05 was considered statistically significant.

## Results

### Excessive mechanical load results in the impairment of mitochondria and dampens desuccinylase SIRT5 expression in NP tissues

**Fig. 1:**
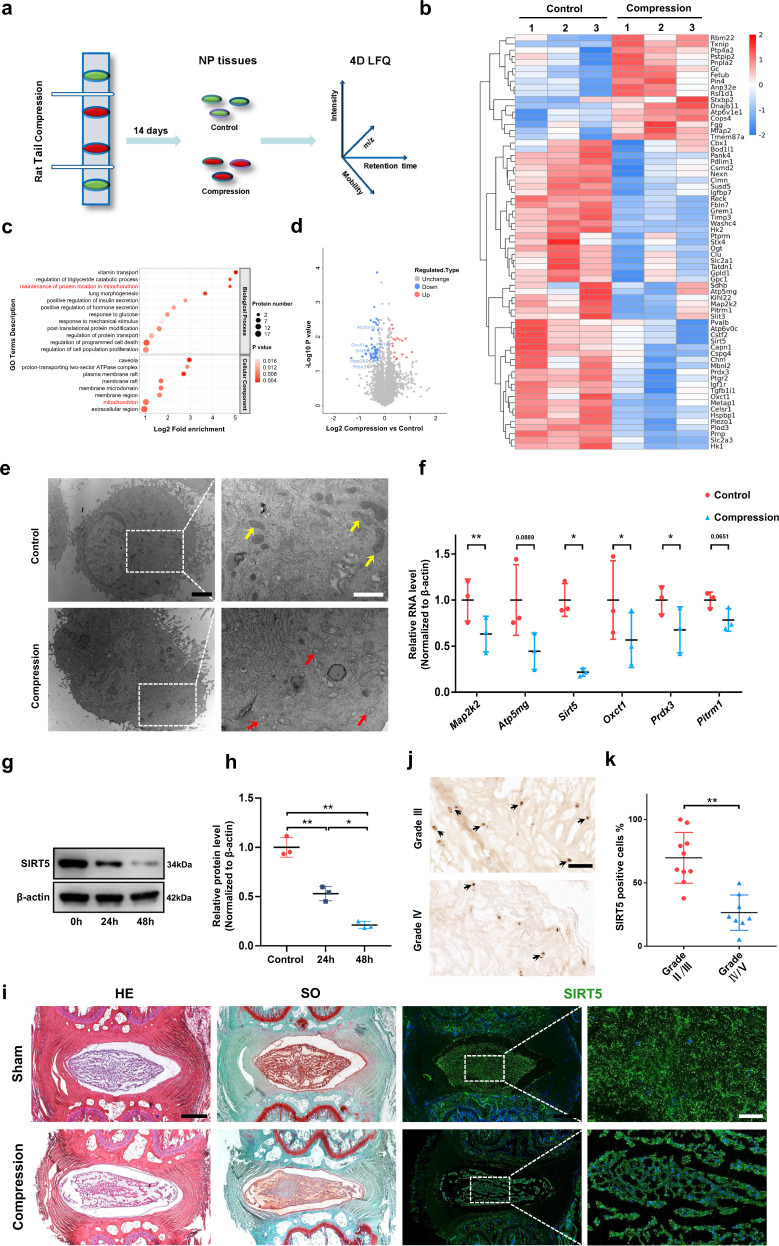
Mechanical stress induces mitochondrial damage and leads to decreased expression of the mitochondrial protein SIRT5 in NP tissues. **a** Experimental scheme of the 4D label-free quantitative proteomics (4D LFQ) of NP tissues harvested from the rat tail compression model 14 days after surgery. White sticks: external fixation points; red oval: compressed NP tissues; green oval: uncompressed NP tissues; *m*/*z*: mass-to-charge ratio. **b** Heatmap depicting the expression profiling of 67 differentially expressed proteins between the control and compressed NP tissues harvested from the indicated IVDs of RCT models (*n* = 3). **c** GO enrichment analysis (biological processes and cellular components) of differentially expressed proteins derived from quantitative proteomics data. **d** Volcano plot of differentially expressed proteins derived from quantitative proteomics data. Mitochondrial proteins are highlighted by gene name. Rat NP cells were cultured in a compression culture chamber and subjected to 1 MPa static compression for 0 h or 24 h. **e** Representative TEM images of mitochondria in NP cells. Yellow arrow: normal mitochondria. Red arrow: unhealthy mitochondria. Black scale bar = 2 μm; white scale bar = 1 μm. **f** *Map2k2*, *Atp5mg*, *Sirt5*, *Oxct1*, *Prdx3*, and *Pitrm1* mRNA levels were determined by qRT‒PCR (*n* = 3). **g**, **h** Western blotting analysis and quantification of SIRT5 protein expression (normalized to β-actin expression, *n* = 3). **i** Representative images of HE, SO, and immunofluorescence staining of SIRT5 in the control and compressed IVDs harvested from the rat tail compression model 14 days after surgery. Black scale bar = 500 μm; white scale bar = 100 μm. **j** Representative IHC staining of SIRT5 in human NP tissues from the Grade II/III (*n* = 10) and Grade IV/V (*n* = 8) groups. SIRT5-positive cells are marked with arrows. Scale bar: 100 μm. **k** Quantification of SIRT5-positive cells in human NP tissues from the Grade II/III (*n* = 10) and Grade IV/V (*n* = 8) groups. Differences between two groups were analyzed by Student’s t test, while the differences among multiple groups were analyzed by one-way ANOVA. The data in the figures represent the mean ± S.D. The *P* value is shown, *P < 0.05, **P < 0.01.

To explore the specific mechanism of mechanical stress-induced NP degeneration, we generated a rat tail compression model in 12-week-old rats. Fourteen days after the operation, IVDs of the compressed and adjacent tail vertebral segments were harvested as the compression group or the control group for 4D label-free quantitative proteomics (Fig. 1a). Among the 2944 quantified proteins, 67 differentially expressed proteins were identified (*p* ≤ 0.05, fold change >1.2) between the control and compression groups: 17 upregulated and 50 downregulated proteins in the compression group (Fig. 1b). Gene Ontology (GO) analysis (Fig. 1c) showed that the differentially expressed proteins were enriched in response to mechanical stimulus and regulation of programmed cell death, demonstrating the successful construction of our in vivo model. More strikingly, GO analysis suggested that mitochondria play an important role in mechanical load-induced degeneration of NP tissues, and compression may affect the localization of proteins in mitochondria. Moreover, subcellular localization (Supplementary Fig. 1a) showed that the expression of some genes encoding mitochondrial proteins, such as *Mapk2k*, *Atp5mg*, *Sirt5*, *Oxc1*, *Prdx3*, and *Pitrm1*, was significantly decreased in the compression group compared with the control group (Fig. 1d). To further validate the proteomics results, we cultured rat NP cells in a compression culture chamber with a static compression of 1.0 MPa for 0 or 24 h. Transmission electron microscopy (TEM) images of the ultrastructure (Fig. 1e) and quantitative analysis of mitochondrial morphology (Supplementary Fig. 1b) showed apparently swollen and fragmented mitochondria within the compression-loaded NP cells compared with the unloaded cells. Moreover, transcriptomic data from the NP cells treated with 1.0 MPa compression showed that one of the top themes of differentially expressed genes focused on mitophagy (Supplementary Fig. 1c), further confirming mitochondrial damage under mechanical stress and the participation of mitochondria in compression-induced IDD. The mRNA levels of the genes encoding differentially expressed mitochondrial proteins were also decreased within NP cells under compression (Fig. 1f), which was consistent with the tendency of proteomic quantification. *Sirt5*, encoding the robust desuccinylase localized in the mitochondria, was the most significantly changed gene under compression. Further Western blotting assays showed that 1.0 MPa compression also significantly inhibited SIRT5 expression in NP cells in a time-dependent manner (Fig. 1g, h). Unsurprisingly, compared with that of the unloaded rat discs, a lower expression of SIRT5 was also observed in mechanically loaded discs, which was confirmed by histological staining and immunofluorescence staining assays (Fig. 1i). To further confirm the relationship between IDD and SIRT5 expression, we collected a total of 18 samples from patients with different Pfirrmann grades of MRI images. Immunohistochemical staining showed a higher proportion of SIRT5-positive cells in sections from the Grade II/III group than in sections from the IV/V group (Fig. 1j, k), indicating a negative relationship between SIRT5 expression and IDD severity. Collectively, these results demonstrated that excessive mechanical load impairs the mitochondria within NP tissue and leads to the decreased expression of the mitochondrial protein SIRT5, suggesting that SIRT5 may play an important role in compression-induced IDD.

### SIRT5 protects NP cells from apoptosis and dysfunction induced by mechanical stress

**Fig. 2:**
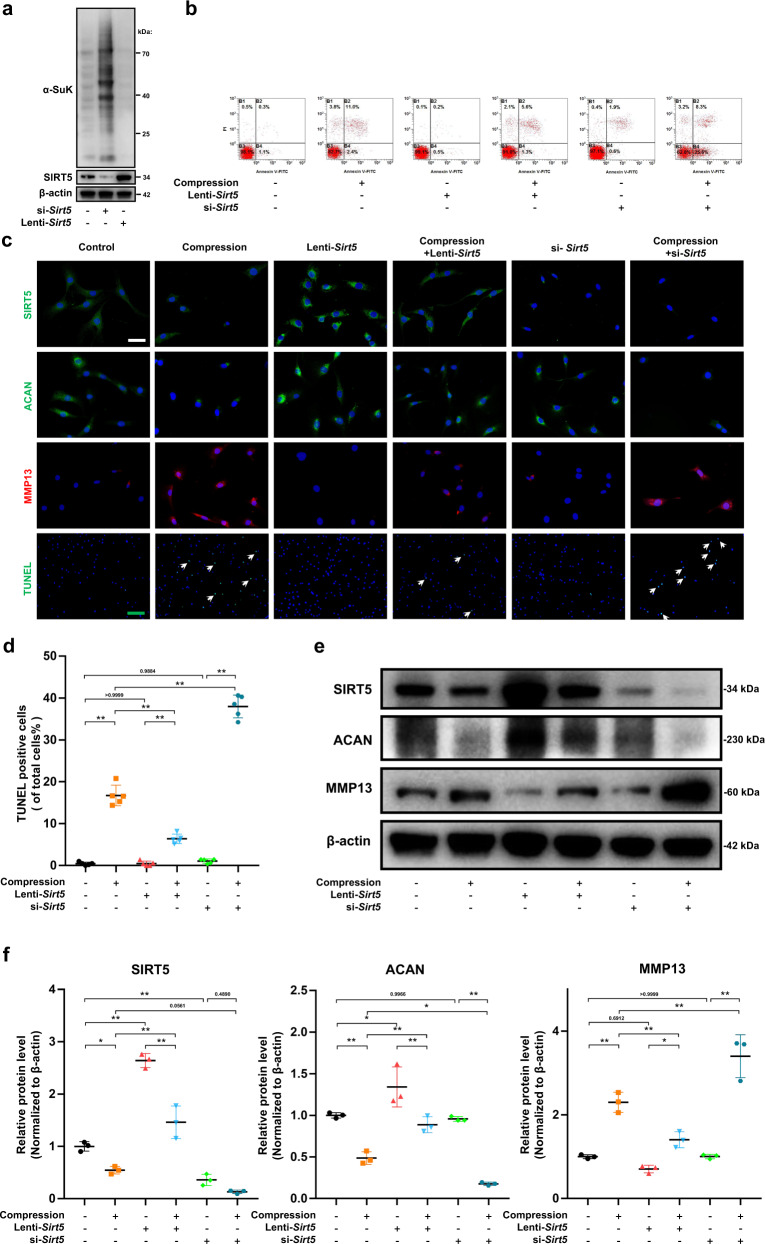
SIRT5 protects NP cells from apoptosis and dysfunction induced by compression. Rat NP cells were cultured with lenti-*Sirt5* or si-*Sirt5* to overexpress or knockdown SIRT5, respectively. **a** Western blotting analysis of protein succinylation and SIRT5 expression (normalized to β-actin expression, *n* = 3). **b** Representative dot plot of cell apoptosis by flow cytometry analysis after Annexin V/PI dual staining (*n* = 3). **c** Representative immunohistochemical staining of the expression of SIRT5, Aggrecan and MMP13 (*n* = 3) and TUNEL staining (*n* = 5) of NP cells. White scale bar = 50 μm; green scale bar = 200 μm. White arrows: TUNEL-positive cells. **d** Quantification of the percentage of TUNEL-positive cells. **e**, **f** Western blotting analysis and quantification of SIRT5, Aggrecan and MMP13 protein expression (normalized to β-actin expression, *n* = 3). Differences among multiple groups were analyzed by one-way ANOVA. The data in the figures represent the mean ± S.D. The *P* value is shown, *P < 0.05, **P < 0.01.

Given the negative correlation between IDD and SIRT5 expression, we hypothesized that SIRT5 plays a protective role in compression-induced disc degeneration. To test this hypothesis, we used *Sirt5* small interfering RNA (si-*Sirt5*) or lentivirus expressing *Sirt5* (Lenti-*Sirt5*) to knock down or overexpress *Sirt5* in NP cells, and their efficiency was confirmed by Western blot assays (Fig. 2a). Given the well-defined function of SIRT5 in desuccinylation, we also detected global protein succinylation within NP cells. Unsurprisingly, a markedly higher level of protein succinylation in the si-*Sirt5* group and a significantly lower level of protein succinylation in the lenti-*Sirt5* group were observed compared with those in the untreated group (Fig. 2a), which further confirmed the high efficiency of siRNA and lentivirus, as well as the potent desuccinylase activity of SIRT5 in NP cells. We next investigated the biological function of SIRT5 in the survival of NP cells. Annexin-V/PI (Fig. 2b) and TUNEL staining (Fig. 2c, d) assays showed that *Sirt5* overexpression significantly attenuated compression-induced NP cell apoptosis, while knockdown of *Sirt5* exacerbated the apoptosis induced by excessive mechanical load. Moreover, immunofluorescence staining (Fig. 2c) and Western blot (Fig. 2e, f) assays showed that *Sirt5* overexpression partially inhibited the catabolic state induced by mechanical stress, including increased aggrecan expression and decreased MMP13 expression, compared with that of the NP cells treated with mechanical stress only. In contrast, knockdown of *Sirt5* aggravated the compression-induced catabolic state. These results proved that the expression of SIRT5 plays a vital role in protecting NP cells from mechanical stress and that SIRT5 is essential for maintaining NP homeostasis in vitro.

### *Sirt5*-deficient mice are more prone to IDD after lumbar spine instability surgery

**Fig. 3:**
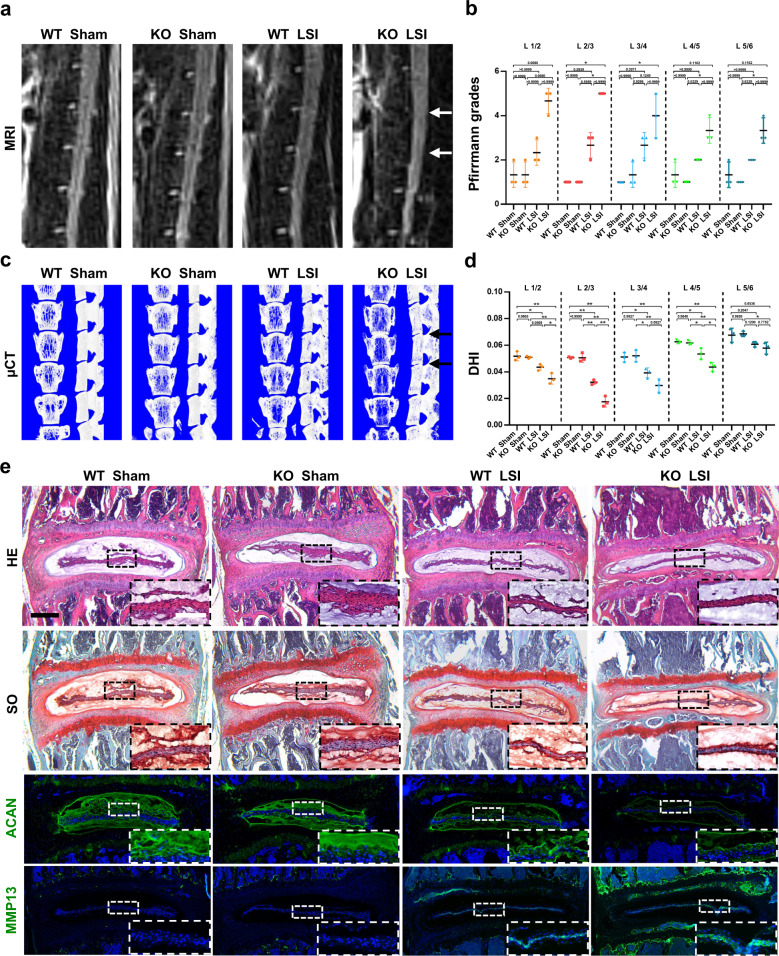
*Sirt5* KO mice exhibit a more severe degenerative phenotype of IVDs after LSI surgery. An LSI model was used to induce IDD in 10-week-old WT and *Sirt5* KO mice. WT sham group, KO sham group, WT LSI group, and KO LSI group (*n* = 3). **a**, **b** Representative MRI scan and statistical graphs of Pfirrmann grades of the mouse lumbar spine (2 months after surgery, *n* = 3). White arrows: L2/3 and L3/4 IVDs. **c**, **d** Representative µCT scans and quantification of DHI of the mouse lumbar spine (2 months after surgery, *n* = 3). Black arrows: L2/3 and L3/4 IVDs. **e** Representative images of HE, SO, and immunofluorescence staining of Aggrecan and MMP13 of the mouse lumbar spine IVDs (2 months after surgery, *n* = 3). Black scale bar = 50 μm; white scale bar = 20 μm. Differences among multiple groups were analyzed by one-way ANOVA, while Pfirrmann grades were analyzed by Kruskal‒Wallis h-tests. The data in the figures represent the mean ± S.D. The *P* value is shown, *P < 0.05, **P < 0.01.

To further validate the protective effect of SIRT5 against mechanical stress in NP tissue in vivo, we performed lumbar spine instability (LSI) or sham surgery on wild-type (WT) mice or *Sirt5* KO mice (WT sham group, KO sham group, WT LSI group, KO LSI group). Two months after surgery, magnetic resonance imaging (MRI) (Fig. 3a) and microcomputed tomography (micro-CT) (Fig. 3c) were performed to evaluate the degree of disc degeneration. The T2-weighted MRI images showed a significantly decreased signal intensity of IVDs in both the WT + LSI group and the KO + LSI group, indicating successful model establishment. Moreover, compared with that of the WT + LSI group, a visibly lower T2 signal intensity of IVDs, especially L2-3 and L3-4 segments of IVDs, was observed in the KO + LSI group, while no difference in T2 signal intensity was observed between the WT sham group and the KO sham group (Fig. 3b), showing faster ECM degradation in NP tissues of the *Sirt5*-deficient mice under sustained mechanical stress. We also analyzed the changes in lumbar disc height of these mice by micro-CT. Three-dimensional reconstructions of the hemisections of lumbar segments showed a more severely decreased disc height index (DHI) in the *Sirt5*-null mice than in the WT mice (Fig. 3d), indicating more severe damage to the disc structure of the *Sirt5*-deficient mice under sustained mechanical load. Consistent with the information obtained by MRI and micro-CT, histological and immunofluorescence staining analyses also showed more severe degenerative changes in NP tissues of the *Sirt5*-null mice caused by LSI surgery compared with the WT mice (Fig. 3e), which were confirmed by increased proteoglycan loss in NP and inner AF tissues, reduced fluorescence intensity of aggrecan and increased fluorescence intensity of MMP13. These findings demonstrated that *Sirt5* KO mice progress to LSI-induced IDD in an accelerated manner, which further confirmed an essential role of SIRT5 in NP tissues against excessive mechanical load. Considering the damaged mitochondria and reduced expression of the mitochondrial protein SIRT5 in NP cells under compression, we speculated that SIRT5 plays a protective role by maintaining mitochondrial homeostasis.

### SIRT5 modulates mitochondrial morphology, composition, and energy metabolism of NP cells

**Fig. 4:**
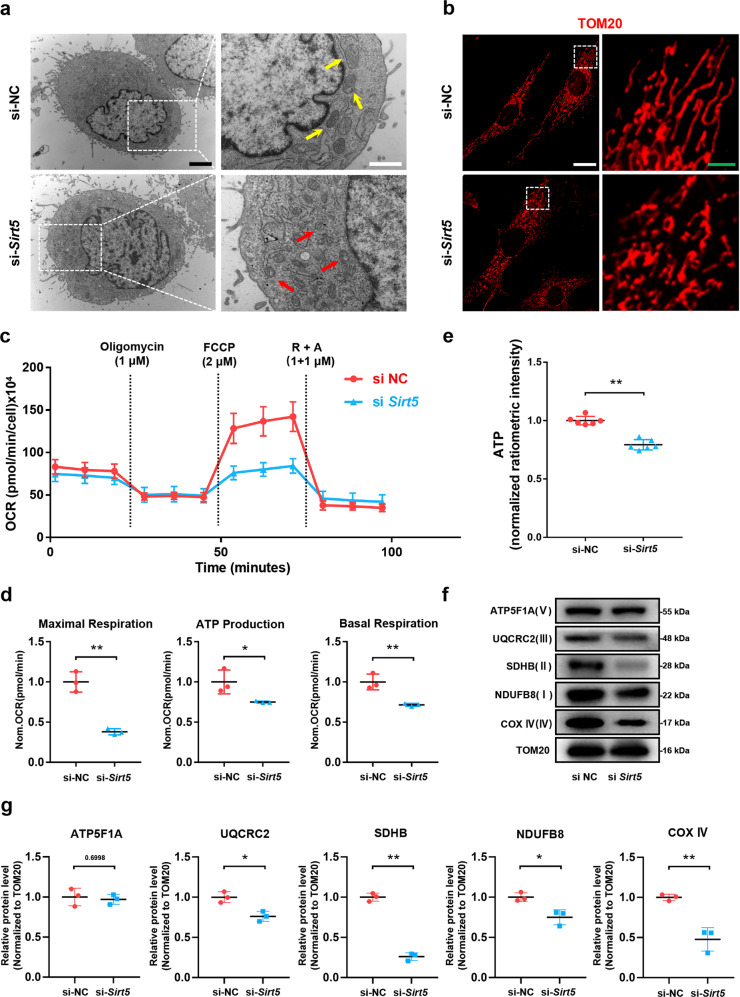
Knockdown of *Sirt5* impairs mitochondrial homeostasis and leads to a decrease in ETC complex subunits in NP cells. **a** Representative TEM images of mitochondri**a** in NP cells from the si-NC and si-*Sirt5* groups (*n* = 3). Yellow arrow: normal mitochondria. Red arrow: unhealthy mitochondria. Black scale bar = 2 μm; white scale bar = 1 μm. **b** Representative immunohistochemical staining of the structure of mitochondria in NP cells from the si-NC and si-*Sirt5* groups (*n* = 3). White scale bar = 50 μm; green scale bar = 10 μm. **c** Seahorse analysis of the oxygen consumption rate (OCR) in the si-NC and si-*Sirt5* NP cells. The OCR was measured continuously throughout the experimental period at baseline and in the presence of the indicated drugs: 1 μM oligomycin, 2 μM FCCP, and 1 μM rotenone with 1 μM antimycin A (R + A). **d** Quantification of maximal respiration, ATP production, and basal respiration of the data from mitochondrial stress tests (*n* = 3). **e** Quantification of ATP levels in NP cells from the si-NC and si-*Sirt5* groups (*n* = 6). **f**, **g** Western blotting analysis and quantification of ATP5F1A, UQCRC2, SDHB, NDUFB8, and COX IV protein expression (normalized to β-actin expression, *n* = 3). Differences between two groups were analyzed by Student’s t test. The data in the figures represent the mean ± S.D. The *P* value is shown, *P < 0.05, **P < 0.01.

To test our hypothesis that SIRT5 plays a protective role in maintaining mitochondrial homeostasis within NP cells, we monitored the mitochondrial morphology, composition, and energy metabolism of NP cells treated with si-*Sirt5* or negative control (si-NC). TEM results (Fig. 4a) and quantitative analysis of mitochondrial morphology (Supplementary Fig. 1d) showed that the vast majority of mitochondria within NP cells exhibited integrated structures under si-NC treatment, whereas the cells treated with si-*Sirt5* showed swollen and fragmented mitochondria. We also performed immunofluorescence staining for TOM20, one of the mitochondrial outer membrane proteins, to visualize mitochondrial morphology within NP cells (Fig. 4b). Similarly, knockdown of *Sirt5* resulted in an increased incidence of fragmented mitochondria. We next detected the effect of SIRT5 on mitochondrial respiration by measuring the oxygen consumption rate (OCR) of NP cells (Fig. 4c). In comparison to the NP cells treated with si-NC, the NP cells treated with si-*Sirt5* exhibited obviously weakened metabolism, including basal respiration, ATP production and especially the maximal respiratory capacity (Fig. 4d), consistent with the decreased ATP levels in the NP cells treated with si-*Sirt5* (Fig. 4e). Moreover, Western blot analysis of mitochondrial proteins showed significantly decreased electron transfer chain (ETC) complex proteins in the si-*Sirt5*-treated NP cells (Fig. 4f, g). Taken together, these results demonstrated that SIRT5 plays an important role in modulating the mitochondrial morphology, composition, and energy metabolism of NP cells, the knockdown of which leads to the impairment of mitochondrial homeostasis.

### SIRT5 regulates the AIFM1-CHCHD4 complex interaction through its desuccinylase activity

**Fig. 5:**
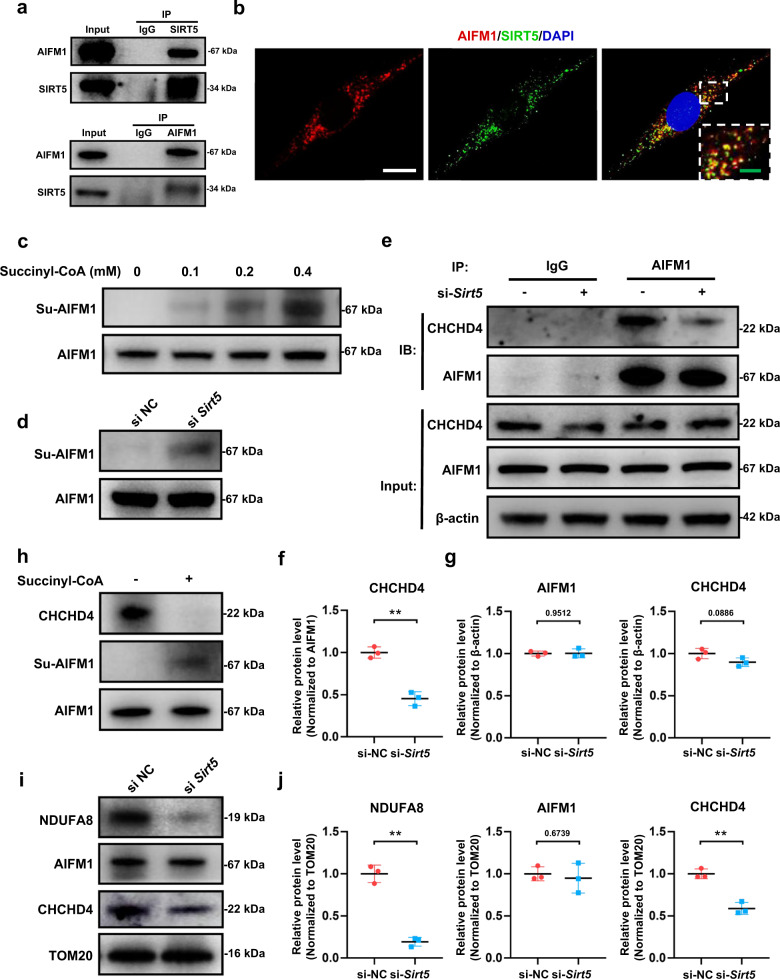
SIRT5 directly binds to AIFM1 and regulates the succinylation modification of AIFM1 through its desuccinylase activity, thereby regulating AIFM1-CHCHD4 complex function. The binding of SIRT5 and AIFM1 was verified in rat NP cells. **a** Co-IP of AIFM1 by SIRT5 antibody and reverse Co-IP of SIRT5 by AIFM1 antibody. **b** Representative immunohistochemical staining of the intracellular location and distribution of AIFM1 and SIRT5. White scale bar = 20 μm; green scale bar = 5 μm. In the in vitro succinylation assay, AIFM1 was purified from NP cells and incubated with the indicated concentrations of succinyl-CoA in vitro. **c** Western blotting analysis of succinylated AIFM1 (*n* = 3). **d** Western blotting analysis of the succinylation of AIFM1 from rat NP cells in the si-NC and si-*Sirt5* groups (*n* = 3). The binding of CHCHD4 and AIFM1 was detected in rat NP cells from the si-NC and si-*Sirt5* groups. **e** Co-IP of CHCHD4 by the AIFM1 antibody. **g** Quantification of CHCHD4 and AIFM1 protein expression (normalized to β-actin expression, *n* = 3). **f** Quantification of CHCHD4 bound to AIFM1 (normalized to AIFM1 expression, *n* = 3). An in vitro succinylation assay was performed to verify the disruptive effect of succinylation modification on the binding between AIFM1 and CHCHD4. **h** Western blotting analysis of succinylated AIFM1 and CHCHD4 protein bound to AIFM1 (*n* = 3). Mitochondrial proteins were extracted from rat NP cells in the si-NC and si-*Sirt5* groups. **i**, **j** Western blotting analysis and quantification of NDUFA8, AIFM1, and CHCHD4 protein expression extracted from mitochondria (normalized to TOM20 expression, *n* = 3). Differences between two groups were analyzed by Student’s t test. The data in the figures represent the mean ± S.D. The *P* value is shown, **P < 0.01.

To explore the specific molecular mechanism of SIRT5 involved in the regulation of mitochondrial homeostasis, we obtained SIRT5-binding protein of NP cells by Co-IP and then identified the binding targets by mass spectrometry (MS) and database bioinformatic analysis. Interestingly, we found that apoptosis-inducing factor mitochondrion-associated 1 (AIFM1)-specific peptides were enriched (Supplementary Table 5), and their combination with SIRT5 was highly credible. AIFM1 ensures the role of CHCHD4 in oxidizing and stabilizing small molecules (including most ETC complex proteins) imported into mitochondria by immobilizing CHCHD4 in the inner mitochondrial membrane. Considering the decreased mitochondrial ETC complex subunits in the *Sirt5* knockdown NP cells (Fig. 4f) and the role of the AIFM1-CHCHD4 complex in importing the intermembrane space components of mitochondria, we hypothesized that SIRT5 affects the function of the AIFM1-CHCHD4 complex. Then, we performed Co-IP and Western blot analyses to verify the MS results. Western blot assays showed that AIFM1 was detected in the Co-IP products of SIRT5, and SIRT5 was also detected in the reverse Co-IP products of AIFM1 (Fig. 5a). Immunofluorescence staining images also showed strong colocalization of SIRT5 and AIFM1 in NP cells (Fig. 5b), confirming the direct interaction between the two molecules. Considering the desuccinylase activity of SIRT5 in NP cells (Fig. 2a) and several potential succinylation sites on AIFM1 lysine residues shown in both Matthew D Hirschey and Eric S. Goetzman’s quantitative mass data^31,32^, we hypothesized that SIRT5 regulates the post-translational modification of AIFM1 through desuccinylation. The results from succinylation assays showed that succinylated AIFM1 in NP cells increased by addition of succinyl-CoA in a dose-dependent manner (Fig. 5c). Western blot results also demonstrated that knockdown of *Sirt5* significantly increased the succinylation of AIFM1 in NP cells (Fig. 5d). Given that succinylation disrupts the electrostatic interactions between proteins, we next detected whether knockdown of *Sirt5* disrupts the interactions between AIFM1 and CHCHD4. Co-IP results showed that knockdown of *Sirt5* had no significant effect on the expression of AIFM1 and CHCHD4 in NP cells (Fig. 5e, g). However, the total quantity of CHCHD4 that bound to AIFM1 was significantly reduced after knockdown of *Sirt5* (Fig. 5e, f). To further verify that SIRT5 abolishes the interaction between AIFM1 and CHCHD4 through its desuccinylase activity, we performed in vitro succinylation assays. After addition of succinyl-CoA, increased succinylation of AIFM1 was observed, accompanied by decreased CHCHD4 that bound to AIFM1 (Fig. 5h). We also detected the protein expression and transportation function of the AIFM1-CHCHD4 complex in mitochondria. Western blot assays showed that the abundance of CHCHD4, not AIFM1, significantly decreased in the mitochondria of the NP cells treated with si-*Sirt5* (Fig. 5i, j). NDUFA8, a subunit of ETC complex I and a well-known substrate of CHCHD4^33^, also decreased in the mitochondria of the NP cells treated with si-*Sirt5* (Fig. 5i, j), further confirming that knockdown of *Sirt5* impairs the activity of the AIFM1-CHCHD4 complex. Taken together, our results demonstrated that decreased SIRT5 expression enhances the succinylation of AIFM1 due to its desuccinylase activity, which in turn abolished the interaction between AIFM1 and CHCHD4 and thus impaired the transportation function of the AIFM1-CHCHD4 complex.

### Excessive mechanical load induces mitochondrial dysfunction and NP degeneration through the SIRT5-AIFM1-CHCHD4 pathway

**Fig. 6:**
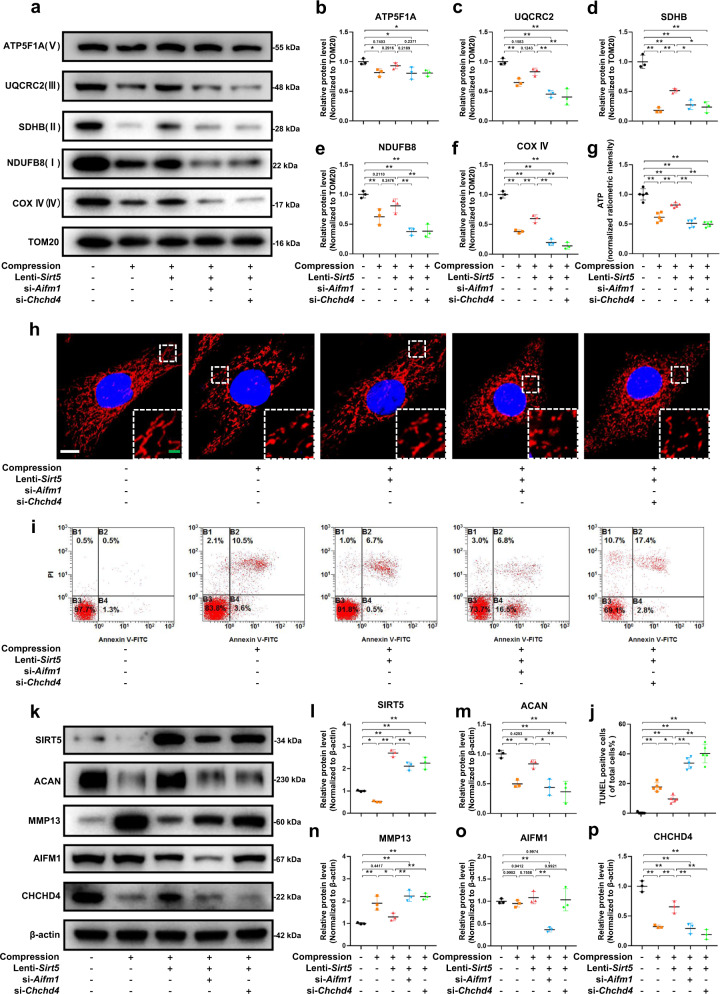
Knockdown of *Aifm1* or *Chchd4* significantly reversed the protective effect of *Sirt5* overexpression on NP cells under compression. Rat NP cells were cultured in a compression culture chamber and subjected to 1 MPa static compression for 0 h or 24 h. Lenti-*Sirt5* and siRNA of *Aifm1* or *Chchd4* were used to verify the protective effect of SIRT5 in rat NP cells under compression. Mitochondrial proteins were extracted from rat NP cells in each group. **a**–**f** Western blotting analysis and quantification of ATP5F1A, UQCRC2, SDHB, NDUFB8, and COX IV protein expression extracted from mitochondria (normalized to TOM20 expression, *n* = 3). **g** Quantification of ATP levels in NP cells in each group (*n* = 5). TOM20 IF staining was performed to visualize mitochondrial morphology. **h** Representative immunohistochemical staining of the mitochondrial structure in NP cells (*n* = 3). White scale bar = 10 μm; green scale bar = 2 μm. **i** Representative dot plot of cell apoptosis by flow cytometry analysis after Annexin V/PI dual staining (*n* = 3). **j** Quantification of the percentage of TUNEL-positive cells (*n* = 5). **k**–**p** Western blotting analysis and quantification of Aggrecan, MMP13, SIRT5, AIFM1, and CHCHD4 protein expression (normalized to β-actin expression, *n* = 3). Differences among multiple groups were analyzed by one-way ANOVA. The data in the figures represent the mean ± S.D. The *P* value is shown, *P < 0.05, **P < 0.01.

To verify the role of SIRT5 and the AIFM1-CHCHD4 complex in mitochondrial dysfunction and NP cell degeneration under mechanical stress, we used *Aifm1-* or *Chchd4*-specific siRNAs to knock down *Aifm1* and *Chchd4* in NP cells, respectively. Western blot assays (Fig. 6a) showed that the expression of ETC complex proteins significantly decreased under mechanical stress. Overexpression of *Sirt5* partially restored the reduction in ETC complex proteins. However, knockdown of Aifm1 or Chchd4 abrogated the rescue effect of SIRT5 under mechanical stress (Fig. 6a–f). Moreover, the morphology of mitochondria in NP cells was damaged under mechanical stress, which was supported by a significantly increased proportion of fragmented mitochondria (Fig. 6h). Overexpression of *Sirt5* partially inhibited the morphological damage to mitochondria, while this effect was abolished by knockdown of *Aifm1* or *Chchd4*. In the same way, knockdown of *Aifm1* or *Chchd4* also abolished the rescue effect of SIRT5 on ATP synthesis under mechanical stress (Fig. 6g). We next performed Annexin-V/PI (Fig. 6i) and TUNEL staining assays (Supplementary Fig. 1e) to detect the role of SIRT5 and the AIFM1-CHCHD4 complex in maintaining NP cell viability under mechanical stress. Similarly, *Sirt5* overexpression inhibited the apoptosis of NP cells induced by compression, while knockdown of *Aifm1* or *Chchd4* reversed this protective effect (Fig. 6i, j). In addition, Western blot assays showed that *Sirt5* overexpression significantly inhibited the degradation of ECM under compression, while knockdown of AIFM1 or CHCHD4 also eliminated this protective effect of SIRT5 (Fig. 6k–p). Overall, our results further confirmed that excessive mechanical load results in mitochondrial dysfunction and NP cell degeneration through the SIRT5-AIFM1-CHCHD4 pathway.

### *Sirt5* overexpression or methylene blue administration effectively alleviates the degenerative phenotype in a compression-induced rat IDD model

**Fig. 7:**
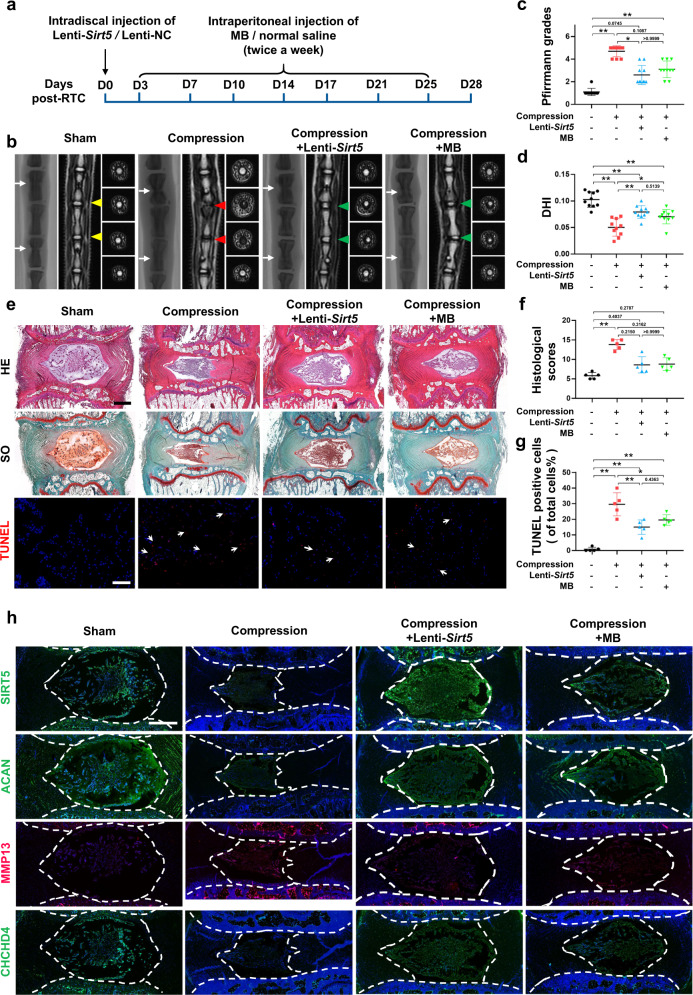
Intradiscal injection of lenti-*Sirt5* or intraperitoneal injection of MB effectively alleviates compression-induced IDD in vivo. RTC was performed in 12-week-old rats, followed by lenti-*Sirt5* or MB treatment. **a** A schematic diagram of the operation. **b** Representative X-ray and MRI images of rat coccygeal vertebrae on Day 28 after surgery (*n* = 5). White arrows: points of injection; yellow triangles: uncompressed discs; red triangles: compressed discs; green triangles: compressed discs with lenti-*Sirt5* or MB treatment. **c** Statistical graphs of Pfirrmann grades (*n* = 10). **d** Statistical graphs of DHI (*n* = 10). **e** Representative HE, SO, and TUNEL staining images. White scale bar: 100 μm; black scale bar: 500 μm. **f** Statistical graphs of histological scores (*n* = 5). **g** Statistical graphs of the percentage of TUNEL-positive cells (*n* = 5). **h** Representative images of immunofluorescence of SIRT5, Aggrecan, MMP13, and CHCHD4. Scale bar: 500 μm. Differences among multiple groups were analyzed by one-way ANOVA, while Pfirrmann grades and histological scores were analyzed by Kruskal‒Wallis h-tests. The data in the figures represent the mean ± S.D. The *P* value is shown, *P < 0.05, **P < 0.01.

To determine whether our above findings have potential translational significance, we performed a rat tail compression model or sham surgery, followed by intradiscal administration of lenti-*Sirt5* or intraperitoneal injection of methylene blue (MB), an alternative mitochondrial electron transfer carrier that could restore mitochondrial dysfunction induced by AIF deficiency (Fig. 7a). MRI images showed that 4 weeks of mechanical load significantly increased the degenerative degree of rat IVDs, while treatment with lenti-*Sirt5* or MB partially restored the elevated Pfirrmann grades induced by static compression (Fig. 7b, c). Radiological images also showed a reduction in disc height in the compression group, which was reversed by both lenti-*sirt5* and MB (Fig. 7d). HE and SO staining revealed that mechanical load led to reduced numbers of NP cells, accompanied by disorganized AF lamellae and collapsed CEPs (Fig. 7e). Unsurprisingly, both lenti-*Sirt5* and MB treatments successfully ameliorated histological damage induced by mechanical stress (Fig. 7f), further confirming the effectiveness of our treatments. Moreover, images from fluorescence microscopy showed increased TUNEL-positive cells (Fig. 7e, g) and MMP13 expression (Fig. 7h) in NP tissue under mechanical stress, accompanied by decreased expression of SIRT5, Aggrecan, and CHCHD4. Both Lenti-*Sirt5* and MB treatments partially reversed the phenotypic changes induced by mechanical stress (Fig. 7h). Taken together, our results further demonstrated the role of the SIRT5-AIFM1-CHCHD4 pathway in regulating IVD homeostasis and confirmed the therapeutic effects of *Sirt5* overexpression or MB administration on the IDD process in vivo.

## Discussion

To a large extent, the current nonoperative treatment (e.g., physical therapy, medication) and surgical treatment (e.g., disc replacement, radiofrequency ablations, and lumbar interbody fusion) targeting IDD aim to alleviate pain and other symptoms. Regrettably, the prevention of IDD and restoration of IVD function is still a pivotal challenge due to the lack of a comprehensive understanding of the mechanisms underlying the occurrence and progression of IDD^34,35^. In this study, we first exploited 4D label-free quantitative proteomics and transcriptomics to analyze the molecular mechanism of IDD induced by mechanical stress and identified the desuccinylase SIRT5 as the core differentially expressed mitochondrial protein. The expression of SIRT5 in the brain was reported to be decreased in neurodegenerative diseases such as Alzheimer’s disease^36^. Furthermore, a model animal demonstrated that *Sirt5* knockout resulted in early osteoarthritic phenotypes compared with wild-type controls, indicating a potential beneficial effect of SIRT5 in degenerative skeletal disorders^37^. However, no study has comprehensively investigated the role of SIRT5 during the development of IDD. Our study is the first report to show that the expression of SIRT5 was negatively correlated with IDD severity induced by mechanical stress. In addition, we showed that SIRT5 is capable of protecting NP cells from apoptosis and dysfunction induced by excessive mechanical load.

As highly dynamic organelles, mitochondria play a major role in the progression of IDD^14^. Mitochondrial shapes are constantly changing through the combined actions of fission, fusion, and motility, which endows cells with the ability to adapt and respond to metabolic or environmental stresses. Fusion contributes to relieving stress by promoting complementation between damaged mitochondria, while fission helps to remove damaged mitochondria and create new mitochondria^7–10^. Therefore, mitochondrial dysfunction significantly damages the ability of NP cells to respond to the ever-changing microenvironment. Kang et al. demonstrated that mechanical stress led to the accumulation of damaged mitochondria and persistent oxidative damage in NP cells^16^. Moreover, the disturbance of mitochondrial homeostasis was observed in degenerated human disc samples. A number of approaches to in vitro or in vivo disc degeneration models impair mitochondrial homeostasis, reduce mitochondrial membrane potential, and increase mitochondrial membrane permeability^14,38^. Our previous study also showed that significant mitochondrial damage could be observed during the development of aging-induced IDD, and the degeneration of the disc could be effectively alleviated by ameliorating mitochondrial damage^5^. Succinylation is a newly recognized post-translational modification that plays a crucial role in mitochondrial homeostasis maintenance^20^. In the present study, we proposed for the first time that succinylation is involved in the regulation of mitochondrial homeostasis within NP cells under excessive mechanical load. We demonstrate that excessive mechanical load promotes succinylation of proteins in NP cells by reducing SIRT5 expression, which in turn leads to disruption of key mitochondrial protein interactions and impairment of mitochondrial protein import and contributes to the development of IDD. However, the potential molecular mechanism of NP cell death caused by mitochondrial dysfunction needs further study.

**Fig. 8:**
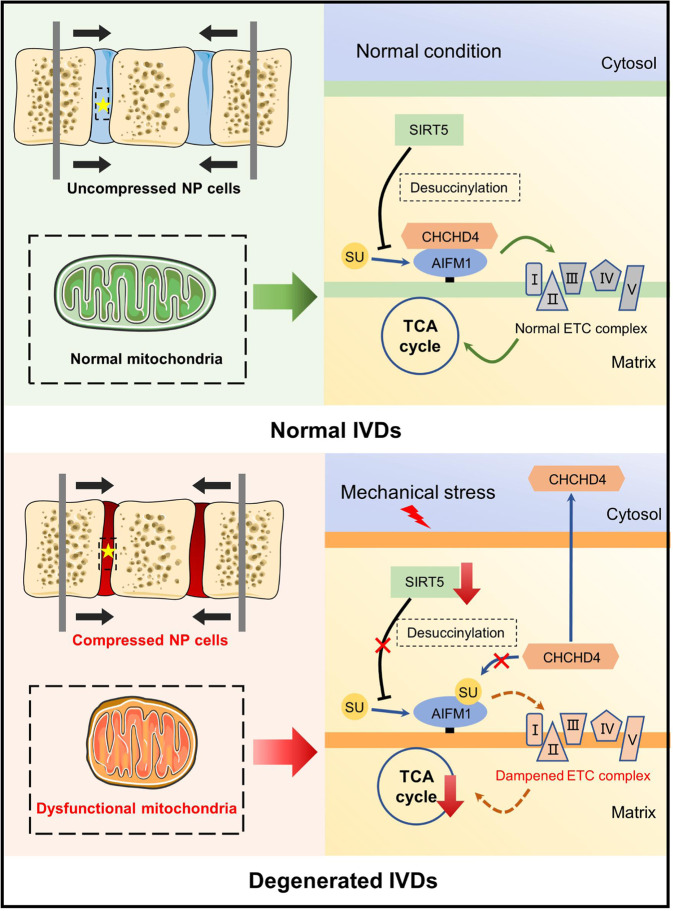
Schematic diagram showing the mechanisms of compression-induced mitochondrial dysfunction within NP cells. Excessive mechanical load increases succinylation of AIFM1 within NP cells by reducing the expression of the desuccinylase SIRT5, which in turn abolished the interaction between AIFM1 and CHCHD4, leading to the disruption of CHCHD4-dependent importation of mitochondrial intermembrane space components and consequent reduction of mitochondrial ETC complex subunits. The reduced ETC complex subunits ultimately lead to mitochondrial dysfunction and contribute to the development of IDD under excessive mechanical load. ETC electron transport chain, IDD intervertebral disc degeneration.

Among the ∼1000 mitochondrial proteins, only a few are encoded by the semiautonomous mitochondrial genome. The vast majority of mitochondrial proteins are encoded by nuclear genes and then must be imported into mitochondria from the cytosol^39^. The AIFM1-CHCHD4 complex plays a vital role in importing the intermembrane space components of mitochondria from the cytosol, including most ETC complex proteins^33,40,41^. As a receptor, CHCHD4 contributes to the correction of folding and stabilization of small molecule substrates through its redox activity^42^. AIFM1, another core protein of the AIFM1-CHCHD4 complex, acts as an anchor, immobilizing CHCHD4 in the inner mitochondrial membrane by directly binding AIFM1. Such binding is indispensable for the prevention of an undesirable escape of CHCHD4 from mitochondria. In addition, either depletion of AIFM1 or disruption of the complex through hypomorphic mutation of AIFM1 results in a decreased abundance of CHCHD4 in mitochondria as well as a reduced import of CHCHD4 substrates, which in turn disrupts ETC complex function and mitochondrial homeostasis^33,40,43^. In the present study, we identified AIFM1 as a novel and crucial downstream target of SIRT5. Under excessive mechanical stress, decreased SIRT5 expression resulted in increased succinylation of AIFM1, which disrupts the electrostatic interaction between AIFM1 and CHCHD4 as succinylation converts the positively charged lysine residues to negatively charged residues (Fig. 8). This role of succinylation modification is further supported by the results of Zhang et al., who showed that succinylation of VLCAD abolished the binding of VLCAD to cardiolipin^44^. Then, disruption of the AIFM1-CHCHD4 complex resulted in the escape of CHCHD4 from the mitochondria, which is supported by the significantly reduced abundance of CHCHD4 in the mitochondria of *Sirt5* knockdown NP cells (Fig. 5i). Moreover, reduced mitochondrial NDUFA8 (Fig. 5i), one of the well-known substrates of CHCHD4^33^, further demonstrated that the increased succinylation of AIFM1, caused by knockdown of *Sirt5*, impairs the activity of the AIFM1-CHCHD4 complex in NP cells.

Our study demonstrated that overexpression of *Sirt5* can effectively lower the catabolism and apoptosis of NP cells induced by excessive mechanical stress in vitro. We also validated the therapeutic effect of intradiscal injection of lenti-*Sirt5* on IDD in a rat tail compression model. The above-mentioned in vitro and in vivo data all indicated that restoration of SIRT5 expression could be a promising therapeutic target for IDD. For a long time, members of the sirtuin family have attracted much attention in a variety of degenerative diseases, including IDD, due to their antiaging properties^45,46^. For instance, promoting the expression of SIRT3 can significantly reduce mitochondrial division in NP cells and promote their antioxidant capacity and mitochondrial dynamics, ultimately delaying IDD under mechanical load or oxidative stress conditions^15,47^. MicroRNA-338-3p aggravates the progression of IDD by reducing SIRT6, a negative regulator of the MAPK/ERK pathway^48^. Moreover, studies have shown that SIRT1 and SIRT2 play a key role in reducing inflammation and oxidative stress, thus delaying the occurrence and progression of IDD^49^. However, since SIRT5 has weak deacetylase activity and no obvious phenotypes were observed in *Sirt5*-null mice under physiological conditions, the function and significance of SIRT5 have not been fully recognized for many years. It was not until the mass exploitation of high-resolution proteomics approaches and advances in bioinformatics that the physiological and pathological significance of SIRT5 came to be the interests of the scientific community. Since SIRT5 is the only known desuccinylase and is primarily located in the mitochondria, the crucial role of SIRT5 in the maintenance of mitochondrial homeostasis is worthy of further investigation^18,20,22^. Given the reduced expression of SIRT5 in a number of degenerative diseases and the satisfying rescue effects from the restoration of SIRT5 in our work and others, targeting SIRT5 therapeutically is of interest. However, to date, no specific activators of *Sirt5* have been successfully developed^18^. Further studies to identify high potency small molecule drugs that specifically target *Sirt5* are warranted.

In addition, the results from our work showed that the decreased SIRT5 impairs mitochondrial importation by interfering with the interactions between AIFM1 and CHCHD4. Determining whether it is possible to alleviate IDD through “bypassing” the broken AIFM1-CHCHD4 complex is logical. Naveen K. Mekala et al. previously showed that MB could fix the impaired mitochondrial integrity and function induced by AIFM1 deficiency through the alternative electron transport effect, effectively alleviating retinal photoreceptor degeneration in Harlequin mice (AIFM1-deficient mice)^50^. Here, we demonstrated that MB can also effectively ameliorate the development of IDD caused by structural disruption of the AIFM1-CHCHD4 complex under compression through its electron transport substitution effect. Moreover, MB is an FDA-approved pharmacological drug and can reduce the severity of discogenic low back pain and postoperative low back pain after lumbar open discectomy via intradiscal injection^51,52^. Therefore, many more clinical studies are expected to further validate the therapeutic effect of MB on IDD patients.

In the present study, we applied excessive mechanical stress to the caudal vertebra of rats directly with an adjustable mechanical loading apparatus. However, due to the limitation of the size of mice and the mechanical properties of materials, it is almost impossible for us to apply stable pressure to the vertebral bodies of mice with conventional pressure devices. Thus, we generated an LSI model, which is widely recognized and applied to simulate abnormal mechanical stress in the intervertebral discs of mice. Hu et al. established an LSI model in mice to mimic IVDD induced by mechanical loading and observed the protective effect of HSP70 on NP cells^15^. Fu et al. also performed an LSI model to evaluate the effect of aberrant spinal mechanical loading on the pathogenesis of IVDD^53^.

However, there are still limitations in this study. First, although strong evidence from several studies has validated murine animals as a good animal model for disc degeneration research, it is inevitable that the biomechanical properties of IVD in neither the rat tail compression model nor the mouse lumbar spine instability model cannot fully phenocopy the deleterious consequences of the excessive mechanical load on humans^54^. More studies using goats or monkeys could provide further insights. Second, in contrast to conditional knockout mice, we cannot rule out the influence of complex phenotypes caused by SIRT5 global knockout mice. Third, we cannot rule out that intraperitoneal injection of MB may affect the function of NP tissue by altering the secretome as well as other unknown mechanisms. Further studies are needed to explore the detailed mechanism of action of MB in vivo. Moreover, the molecular mechanism of compression-induced reduction of SIRT5 expression in NP cells is also worthy of further investigation.

In summary, our study reveals a novel molecular mechanism of disc degeneration induced by mechanical stress. Our results demonstrated, for the first time, that excessive mechanical load increases succinylation levels in NP cells by reducing the expression of the desuccinylase SIRT5, impairing mitochondrial function and contributing to the subsequent occurrence of IDD. Furthermore, we demonstrated that increased succinylation of AIFM1 leads to the dissociation of AIFM1 and CHCHD4 and subsequent reduction in the ETC complex and mitochondrial dysfunction. Finally, we evaluated the effectiveness of two different therapeutic approaches targeting disrupted mitochondrial protein importation under excessive mechanical load in a rat tail compression model by upregulating *Sirt5* expression or MB administration. Overall, our study provides new insights into the occurrence and development of IDD and offers promising therapeutic approaches for IDD.

## Supplementary information

Supplementary figure and tables

Supplementary Table 5. IP-MS dataset

## Data Availability

The datasets used and/or analyzed during the current study are available from the corresponding author on reasonable request.
