# Supplementary material for: SIRT5-related desuccinylation modification of AIFM1 protects against compression-induced intervertebral disc degeneration by regulating mitochondrial homeostasis
Source: Exp Mol Med. 2023 Jan 18;55(1):253–68. doi: 10.1038/s12276-023-00928-y (PMC9898264; doi:10.1038/s12276-023-00928-y)
Supplement: Supplementary file 1 — Supplementary figure and tables [file 12276_2023_928_MOESM1_ESM.pdf]

Supplementary figure and tables

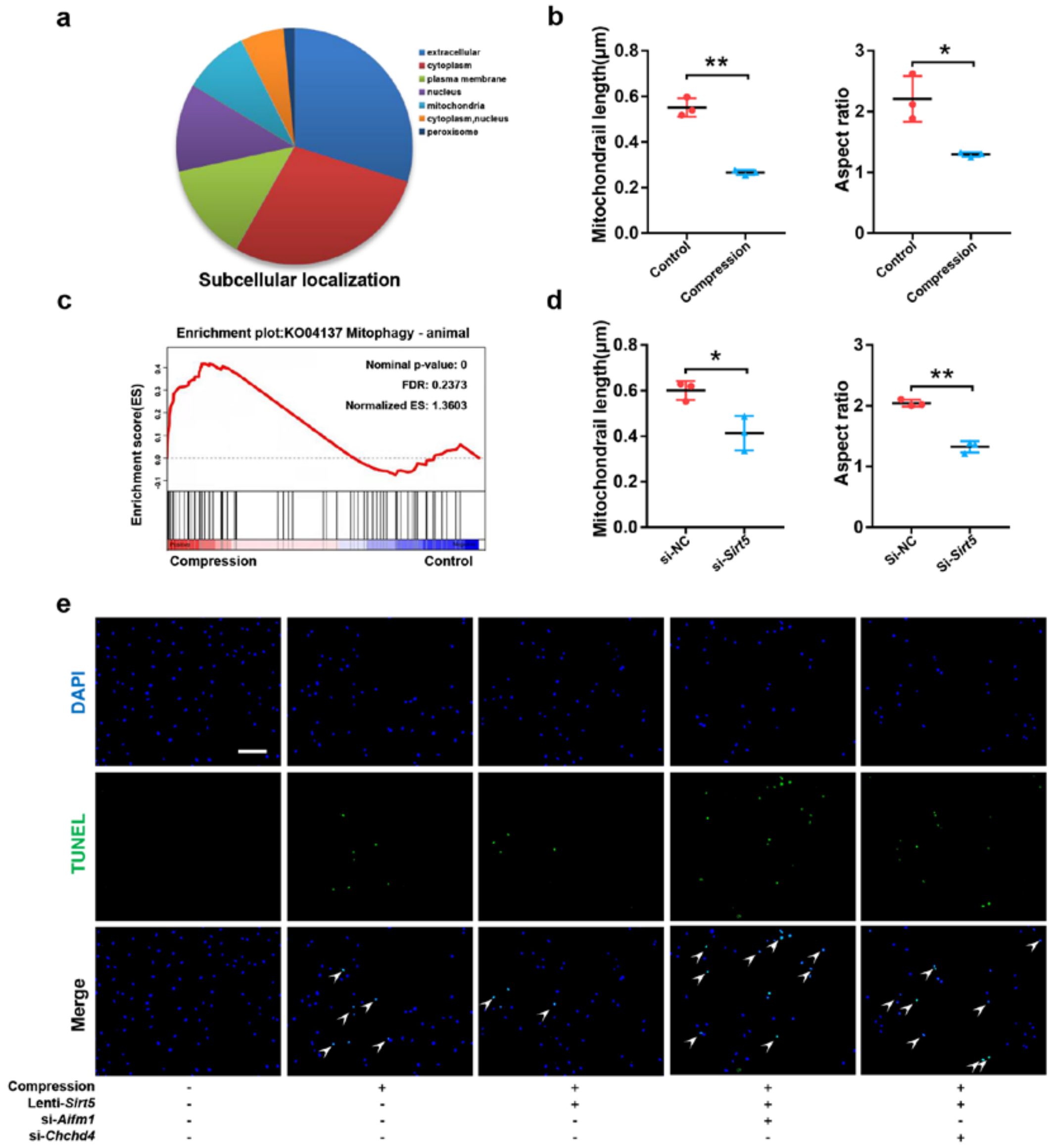

Supplementary Fig. 1 a Subcellular localization of differentially expressed proteins derived from quantitative proteomics data. RNAseq analysis was performed in rat NP cells. b d Quantitative analysis of mitochondrial length and aspect ratio within NP cells (the average value of all mitochondria in the cell) among groups as indicated(n=3). c KEGG showed that mitophagy was enriched in NP cells under compression. e TUNEL staining of NP cells. Scale bar: 100 μm (n = 5). The data in the figures represent the mean ± S.D. *$P < 0.05$, **$P < 0.01$.

Supplementary Table 1. Demographic data of patients.

Patients NO.	Age	Gender	Level	Pfirrmann grading
**Grade II/III group**				
1	45	M	L3/4	2
2	37	M	L4/5	3
3	51	F	L3/4	3
4	53	F	L4/5	2
5	34	F	L5/S1	2
6	57	M	L4/5	3
7	65	F	L3/4	3
8	43	M	L3/4	3
9	53	F	L4/5	3
10	29	M	L4/5	2
**Grade IV/V group**				
11	34	M	L5/S1	4
12	61	F	L4/5	4
13	42	F	L5/S1	5
14	59	F	L3/4	4
15	72	M	L5/S1	5
16	68	F	L4/5	5
17	54	M	L4/5	5
18	38	F	L4/5	4

Supplementary Table 2. primers of Genotype

Primer	Sequence (5’→3’)	Primer Type
Sirt5 Ko Mut	TGCCTGGGGTTGGGGTGTG	Forward
	TGGTGGGGTCTGTGGGAGGAAC	Reverse
Sirt5 Ko Wt	CCAGTGGCAGGAAGAGC	Forward
	TGAGCCACATTGAACACAT	Reverse
Genotype	Ko=402bp Wt=605bp	

Supplementary Table 3. Sequences of siRNA

Name	sense (5'-3')	antisense (5'-3')
si-*Sirt5*	GCAAUGUUGCUGAGAACUATT	UAGUUCUCAGCAACAUUGCTT
si-*Aifm1*	GGCUGGAAAUAGAUUCCGATT	UCGGAAUCUAUUUCCAGCCTT
si-*Chchd4*	GGAAGGAUCGAAUCAUAUUTT	AAUAUGAUUCGAUCCUUCCTT

Supplementary Table 4. Sequences of primers used for qRT-PCR

Gene (Rat)	Sequence (5′–3′)	
Sirt5	Forward	CCTTTTGCAGCCTGCCC
	Reverse	TGCGTTCGCAAAACACTTCC
Atp5mg	Forward	AAACGTGGCATTGTTGGCTA
	Reverse	CAGTGTAACAAAGCCAAAGACGA
Map2k2	Forward	TGCCAAGGAACTAGAGGCCA
	Reverse	CTGTCCATCCCATGACCACTG
Oxct1	Forward	TGTGCCTGCTACTTTTCCGT
	Reverse	CACAACCCGAAACCACCAAC
Prdx3	Forward	CACTCCGTTTGGTAAAGGCG
	Reverse	TGTTGGACTTGGCTTGATCGTA
Pitrm1	Forward	TACTGGGACAAGCCTAGGGA
	Reverse	CAAAACCTTTCTCTGGTAAGAGG
